# Age, Sex, and Race/Ethnicity Associations between Fat Mass and Lean Mass with Bone Mineral Density: NHANES Data

**DOI:** 10.3390/ijerph182312606

**Published:** 2021-11-30

**Authors:** Meghan E. Garvey, Ling Shi, Philimon N. Gona, Philip J. Troped, Sarah M. Camhi

**Affiliations:** 1 Garvey Wellness Corp, 9 Temple Place, Andover, MA 01810, USA; 2 Department of Biology, Simmons University, 300 the Fenway, Boston, MA 02115, USA; 3 Department of Nursing, University of Massachusetts Boston, 100 Morrissey Blvd, Boston, MA 02125, USA; Ling.Shi@umb.edu (L.S.); Phil.Troped@umb.edu (P.J.T.); 4 Department of Exercise and Health Sciences, University of Massachusetts Boston, 100 Morrissey Blvd, Boston, MA 02125, USA; Phil.Gona@umb.edu; 5 Department of Kinesiology, University of San Francisco, 2130 Fulton St, San Francisco, CA 94117, USA; scamhi2@usfca.edu

**Keywords:** bone health, obesity, osteoporosis, body composition

## Abstract

Rising rates of obesity and osteoporosis have public health implications; hence, understanding the relationships between body composition (fat mass (FM) and lean mass (LM)) and bone mineral density (BMD) is important. The purpose of this study is to investigate these associations in a large representative sample. A cross-sectional analysis was conducted using National Health and Nutrition Examination Survey participants (*n* = 1717, age 44.1 ± 14.2 years) who had complete dual energy x-ray absorptiometry (total BMD g/cm^2^, FM kg, and LM kg) and covariate data. Hierarchical linear regression models were fitted, controlling for demographic and behavioral covariates. Stratum-specific models were fitted by race, sex, and age group. Significant negative associations were found for FM and BMD (β = −0.003) and significant positive associations for LM and BMD (β = 0.007). Stratum-specific analyses by race were consistent between groups, while variations in negative association magnitudes were seen in FM for sex (males β = −0.005 vs. females β = −0.002) and age (under 45 years of age β = −0.005 vs. 45 years and older β = −0.002). Consistent positive linear associations in total and stratum-specified models between LM and BMD could suggest a potential mechanical influence on bone health. The biological mechanisms driving the magnitude variations between FM and BMD by sex and age require more investigation.

## 1. Introduction

Obesity, defined as a body mass index (BMI) >30 kg/m^2^, is a growing public health concern in the United States [1]. While the causal relationships between obesity and several comorbidities are understood, the relationship between obesity and bone health remains uncertain.

Commonalities between obesity and osteoporosis exist in genetic predisposition and common progenitor cells, laying the foundation for a significant, yet complex effect modification between fat and bone [2]. Historically, increased body mass has been linked to an increase in bone mineral density (BMD) and was believed to be a protective factor against osteoporosis [3]; however, body mass may not explain the nuances of body composition. Two major subcomponents are fat mass (FM) and lean mass (LM). While the current literature agrees that a positive relationship exists between LM and BMD, associations between FM and BMD vary. Some studies have linked FM/obesity to lower BMD per unit BMI [4,5], while others have found FM to be positively associated with BMD [3]. Among the limitations of population-based studies investigating BMD are the frequent exclusion of people younger than 50 years of age and the lack of data on potential racial differences. Investigations into the variations in the associations between FM and LM with BMD have been lacking, impacting risk stratification for BMD-related conditions for these sub-groups. Based on U.S. data for the period 2008–2014, Black women were less likely to be screened for bone health conditions than their white counterparts, even when one or more comorbidities were present [6]. Therefore, the purpose was to investigate the association between FM and LM with BMD in a nationally representative study and examine how these relationships vary by sex, age groups, and race/ethnicity.

## 2. Materials and Methods

Pooled data from the National Health and Nutrition Examination Survey (NHANES—years 2003–2004 and 2005–2006) participants (*n* = 1717, 46.4 ± 13.7 years old, 47.7% female) were included in this cross-sectional analysis. NHANES uses a complex sampling and weighting scheme to obtain a representative sample of the U.S. population. Detailed protocols have been previously published by the Centers for Disease Control and Prevention, National Center for Health Statistics [7]. The University of Massachusetts Boston Institutional Review Board approved the study (code FWA00004634 and date of approval 01 August 2018). 

Participants were included in the analysis if they had undergone dual energy x-ray absorptiometry (DXA) and had complete data for age, sex, race/ethnicity, calcium intake, physical activity, and current smoking status. For the analysis, participants who indicated they were white, Black, or Mexican American were included; all other race/ethnicities were excluded due to low numbers of participants after other exclusions (*n* = 59). Participants were excluded if they were pregnant or lactating (*n* = 566) or had a history of glucocorticoid use (*n* = 104). 

### 2.1. Body Composition

Components of bone (total BMD g/cm^2^) and body composition (FM kg and LM kg) were measured using DXA scans (Hologic QDR 4500A, Hologic Inc., Bedford, MA, USA).

### 2.2. Anthropometric Measurements

A Seca (Chino, CA, USA) electronic fixed stadiometer with a vertical backboard and a moveable headboard was used to measure standing height. A Toledo (Columbus, OH, USA) electronic weight scale was used to measure the body weight of participants. Height and weight were measured by trained technicians. BMI was calculated by dividing weight in kilograms (kg) by height in meters (m) squared. The NHANES Anthropometric Procedure Manual describes the measurement protocols, equipment, and quality control in detail [7].

### 2.3. Demographic and Behavioral Covariates

Demographic and behavioral information such as sex, race/ethnicity, age, menopausal status, and smoking status of the participants were collected in the home via questionnaires administered by trained interviewers using a computer-assisted personal interviewing (CAPI) system. The NHANES dietary interview component was conducted in partnership with the United States Department of Agriculture (USDA) and the United States Department of Health and Human Services (HHS) and is called “What We Eat in America” (WWEIA). The nutritional survey sample design and all aspects of data collection were provided by HHS’ National Center for Health Statistics (NCHS), while the USDA’s Food Surveys Research Group (FSRG) had responsibility for the dietary data collection methodology and database maintenance. Physical activity (PA) was assessed via the ActiGraph AM7164 monitor, (ActiGraph (Ft. Walton Beach, FL, USA)). Valid wear days were defined as ≥10 h of wear time [8], and participants were included in the analysis if they had at least 5 out of 7 valid wear days. Each minute of wear time was classified using established cut points into moderate activity (≥2020 counts) and vigorous activity (>5999 counts) [9].

### 2.4. Statistical Analysis

Descriptive statistics, including means, standard deviations (SD), numbers, and percentages were computed for all measures. Exploratory data analysis conducted before the statistical analysis revealed that FM and LM could appear in the same model without risk of collinearity (mean VIF = 1.02). 

The sample was stratified by the following factors: age group, sex, and race/ethnicity. Age groups were determined by taking into consideration the fact that peak bone mass is accrued by the age of 30 years [10] and also the effects of late reproductive hormonal changes [11]. To address the paramount differences between menopausal women and older men, menopausal women were further stratified to properly account for hormonal variations between them and older males. Sex was categorized as male or female and race/ethnicity as white, Black, or Mexican American.

The variations between stratification groups for BMD, FM, LM, and key covariates were determined through ANOVA analysis. A Bonferroni correction was used to account for multiplicity. The dependent variable in this analysis was BMD (g/cm^2^). The associations between FM and LM (independent variables) were assessed using regression coefficients for FM and LM. Hierarchical regression models were built to explore these relationships: model 1 was adjusted for age and sex only, model 2 was additionally adjusted for demographic covariates (age, sex, race, and height), as well as for behavioral covariates (smoking status, daily calcium intake, and MVPA), and model 3 additionally included significant interaction terms (FM*age, FM*sex, and LM*age). 

Model 3 was utilized to assess whether there was heterogeneity across age groups and between men and women. Interaction terms for age group and sex with FM and with LM were included in the ANCOVA models. All interaction terms attained statistical significance (*p* < 0.05), suggesting the presence of heterogeneity in age group and sex with each of FM and LM. To account for the heterogeneity indicated by significant sex and age-group interactions with LM and FM, sex- and age-specific (20–44 years old and 45+ years old) regression models were fitted. The model for females was additionally adjusted for menopausal status. Adjusted coefficients of determination, R^2^, were compared to determine model fit and quantify the proportion of variance in BMD explained by the covariates included in each model. 

The statistical analysis accounted for the complex study design of NHANES, i.e., unequal sampling weights, strata, and clustering were accounted for in the statistical models. The statistical analysis was conducted using Stata 15.0 (StataCorp, College Station, TX, USA). A *p*-value < 0.05 was considered statistically significant.

## 3. Results

A descriptive analysis of the study sample (*n* = 1717; age 44.1 ± 14.2 years; 47.7% female) can be found in Table 1a; 52.1% were white, 23.7% were Black, and 24.2% were Mexican American. 

Mean height, weight, lean mass, fat mass, BMI, total BMD, calcium intake, and MVPA per week were significantly different between males and females, but age was not (Table 1a). Total BMD was substantially statistically different between the two age groups (all *p* < 0.03) (Table 1c). Between race/ethnicity groups age, height, weight, BMI, fat mass, calcium intake, and MPVA per week were significantly different (Table 1b).

As shown in Table 2, FM was negatively associated with BMD (*p* < 0.001), and LM was positively associated with BMD (*p* < 0.001) in all three models (see Table 2). The beta regression coefficients for FM and LM changed little from model 1 to model 3. The adjusted coefficient of determination, R^2^, increased from 0.33 in model 1 to 0.37 for model 3.

The results of the summary linear regression coefficients for the stratified analyses are shown in Table 3. Beta regression coefficients for FM become more negative for individuals 45 years and older; however, for menopausal individuals the association becomes non-significant (Table 3). When stratified by race, the beta regression coefficients are the same for FM across the three groups and similar for LM across groups (Table 3). 

## 4. Discussion

In the present study, our results show consistent negative associations between FM and BMD and positive associations between LM and BMD, with attenuation of effects when adjusting for both demographic and behavioral covariates. FM regression coefficients, on the other hand, were attenuated in the stratum-specific analyses, particularly in females and individuals between the ages of 20 and 44 years. The association between FM and BMD becomes non-significant in menopausal females. Associations between both FM and LM, and BMD did not vary by racial/ethnic group. 

Historically, obesity was viewed as an osteoprotective agent, yet more recent literature has indicated that the relationship could be more complex [5]. With the escalating prevalence of osteoporosis diagnoses [12], the increasing prevalence of obesity [1], and inconsistent study findings, it is imperative to analyze the body composition components of FM and LM rather than the total body mass in relation to BMD. The findings in our study suggest that increased body weight would be osteoprotective only with a higher LM vs. FM content. These findings are consistent with others in the field, such as those of [5], who indicated that although higher body mass was associated with a higher absolute BMD, site BMD per kg of weight was negatively associated and obesity appeared to be a risk factor for prevalent vertebral fractures [5]. Although this contradicts other studies, such as that of [13] indicating a positive relationship between FM and BMD [13], the findings are similar to studies such as that of [3], where positive associations between FM and BMD were found only above 40 kg of FM [3]. The complex nature of the relationships between FM and BMD highlights the need for further investigation into the mechanism of action driving these associations, to uncover any variables that may be mediating those associations in different samples.

The overall relationship between FM and LM, and BMD has been more heavily debated in the past three decades, particularly due to the inconsistent findings for FM. Most notably, the relationship between LM and bone is theorized to stem from evidence that muscular contractions impact the growth of bone in combination with other factors, such as gravitational force [14]. The positive association between LM and BMD found in this study was consistent within the study sample as well as in sub-groups defined by sex, age, and race/ethnicity. Our findings support previous findings that LM contributes to bone health [14] and could suggest that this relationship is independent of sex-related and other age-related factors. 

While FM’s association does remain consistently negative in our analyses, its regression coefficient effect varies more between conditions than is observed with LM. This observation, specifically between models 2 and 3 and between the age- and sex-specific analyses, supports conclusions from other researchers indicating that the mechanism behind the relationship between FM and bone health is complex [2]. 

It has been reported that osteoporosis and obesity share common genetic and hormonal risk factors [2]. These commonalities could potentially be driving the heterogeneity in the relationship between FM and BMD noted in our findings. Recent research has noted that adipocytes and osteoblasts originate from a common progenitor cell and that adipokines and hormones are both involved in the regulation of FM and bone remodeling [15]. Evidence also suggests that increased FM is associated with poor bone quality and increased risk of fracture [4,5]. Other studies have found that although the absolute BMD is greater in obese individuals, various site BMDs per kg of body mass were lower for overweight and obese individuals with BMI > 25 kg/m^2^ vs. normal weight (BMI between 18 and 24.9 kg/m^2^) [5]. It was also observed in females that for those with a BMI between 18.5 and 29.9 kg/m^2^, a negative association between FM and BMD was present; the association became positive for females with a BMI of 30 or greater [3]. While our study did not see any positive associations between FM and BMD, and analyses by BMI categories were not conducted, the effect modification of FM and sex was examined. When the interaction terms were included in the regression model its negative effect size increased. Looking at the interaction between the two independent variables of FM and sex, this suggests that for every kg of FM females have a BMD 0.002 g/cm^2^ higher than males, and in each age group BMD is decreased by 0.0002 g/cm^2^. While this may not reflect the positive relationship that [3] found between FM and BMD in females, it may reflect a more robust negative relationship in males, which is noted when the models are analyzed separately by sex [3]. The magnitude of the coefficient increased in males, while it decreased in females. 

Mechanostatic theory indicates that bone remodeling is greatly influenced by the mechanical loads applied to it [16]. This has been found to include strain forces, forces applied with the contraction of skeletal muscle, and gravitational forces, i.e., forces applied through body mass [17]. While PA was adjusted for in our model, it is important to investigate the independent effect of PA on BMD. For the current study, when considering body composition, the relationship of PA to both the LM and FM independent variables was explored (r = 0.22 and −0.29, respectively, both *p* < 0.001) leading to the decision to adjust for PA in our models. 

It is important to note, when considering our findings, that clinically significant changes in BMD are not determined by fixed increments. Due to the normal variations between age, sex, and race/ethnicity [18], absolute changes in BMD of 1.7–2% have been found to be clinically significant [19,20] also determined that a 2% gradient-of-risk beta for increased marrow fat content significantly impacted the relative risk of fracture [20].

While coefficient differences were noted in other stratum-specific analyses, our findings were consistent between all race/ethnicity groups. Studies focusing on BMD differences and fracture-risk stratification between racial/ethnic groups is not new to the literature; however, it is important to note that the effects of LM and FM on BMD remain consistent regardless of race. A future aim of research should be to investigate the contributory factors driving these differences, such as ancestral history and variations in bone microarchitecture [21]. This study’s findings may have important clinical implications as more research is conducted. Currently, bone health screening is influenced by race, regardless of comorbidities [6]. If the negative relationships between fat mass and BMD are consistent between race/ethnicity, comorbidities such as having a higher fat mass should be utilized as more significant driving factors in bone health risk stratification. 

Although additional studies are warranted, the variations between the age- and sex-stratified models found may indicate the greater importance of the location of fat deposits in the body [22], as body composition varies between sex and age [23]. For equivalent BMIs, females across the adult lifespan have significantly more FM compared to males [23]. Additionally, it has been noted that obesity plays a role in bone metabolism driven by the biochemical properties of FM, which vary between sex, as we age, and with overall body composition changes [20,24] articulates a few factors driving the interrelated relationship, including proinflammatory cytokines, which are elevated with obesity, increasing bone reabsorption. A decrease in bone marrow osteoclastogenesis is also associated with aging, accompanied by an increase in bone marrow adipogenesis [24]. These mechanisms of action should be the focus of future research into these associations.

A major strength of our study is the large, diverse, population-based sample [25], and to the best of our knowledge this is one of the few studies to focus on the associations between body composition (LM and FM) and BMD by race in a large, population-based sample. The use of gold-standard measurement tools such as DXA for body composition (LM, FM, and BMD) measurement and objective assessment of physical activity with accelerometers are additional strengths of this study.

Among the limitations of the present study is the cross-sectional and observational design of NHANES which precludes inference on causality between FM or LM and bone. Another limitation is the fact that key covariates such as smoking history and daily calcium intake were acquired by questionnaire, potentially introducing bias. This study also did not include bone microarchitecture or site-specific measurements of BMD in its analyses; including these in future studies would be important to confirm the negative associations found in the present study for FM and BMD in this sample. 

## 5. Conclusions

We found that LM consistently showed a positive association with BMD, which could be suggestive of a systemic effect of LM on bone health. The magnitude of the associations between FM and BMD varied between sex and age groups but not between race/ethnicity groups; the exact mechanisms driving these associations, however, require further investigation. With the increasing prevalence of obesity in the U.S. over the past 20 years and its increased burden on the healthcare system, emphasis should be placed on lean mass accrual across all ages, races, and sexes, and on the risks of FM accumulation, particularly in males. This may be indicative in the promotion of favorable bone health.

## Figures and Tables

**Table 1:** (a) Characteristics of participants: overall and by sex. (b) Characteristics of participants by race/ethnicity. (c) Characteristics of participants by age categories.

**(a)**				
		**Overall**	**Female**	**Male**
**Variable**		***n* = 1717**	***n* = 819 (47.7%)**	***n* = 898 (52.3%)**
Age		46.4 (±13.7)	46.9 (±13.7)	46.0 (±13.8)
	20–44 years	744 (43.3%)	342 (41.8%)	402 (44.8%)
	45+ years	973 (56.7%)	477 (58.2%)	496 (55.2%)
Race/Ethnicity				
	White	895 (52.1%)	420 (51.3%)	475 (52.9%)
	Black	407 (23.7%)	208 (25.4%)	199 (22.2%)
	Mexican American	415 (24.2%)	191 (23.3%)	224 (24.9%)
Height (cm)		168.9 (±9.8)	162 (±6.7)	175.2 (±7.6) **
Weight (kg)		81.4 (±17.4)	75.9 (±16.9)	86.4 (±16.3) **
BMI (kg/m^2^)		28.5 (±5.7)	28.9 (±6.3)	28.1 (±4.9) *
	Underweight & Normal <25	490 (28.5%)	260 (31.8%)	230 (25.6%)
	Overweight BMI 25–29.9	624 (36.3%)	237 (28.9%)	387 (43.1%)
	Obese BMI ≥ 30	605 (35.2%)	322 (39.3%)	281 (31.3%)
Lean Mass (kg)		51.7 (±11.5)	42.9 (±7.1)	59.7 (±8.5) **
Fat Mass (kg)		28.0 (±10.5)	31.4 (±10.9)	24.8 (±9.2) **
BMD (g/cm^2^)		1.17 (±0.12)	1.1 (±0.11)	1.2 (±0.11) **
Calcium intake (mg/dL)		1851.3 (±926.5)	1670.9 (±811.2)	2015.9 (±992.5) **
Smoking				
	Current Smoker	388 (22.6%)	136 (16.6%)	252 (28.1%)
MVPA min per week		145.4 (±119.6)	109.1 (±96.3)	178.6 (±128.8) **
**(b)**				
		**White**	**Black**	**Mexican American**
**Variable**		***n* = 895 (52.1%)**	***n* = 407 (23.7%)**	***n* = 415 (24.2%)**
Age		47.3 (±13.2)	47.0 (±13.9)	43.9 (±14.3) **
	20–44 years	334 (38.4%)	172 (42.3%)	228 (54.9%)
	45+ years	551 (61.6%)	235 (57.7%)	187 (45.1%)
Height (cm)		170.9 (±9.5)	169.3 (±9.4)	164.1 (±8.9) **
Weight (kg)		82.4 (±18.5)	84.0 (±17.0)	76.6 (±15.3) **
BMI (kg/m^2^)		28.2 (±5.8)	29.4 (±5.9)	28.4 (±5.0) **
	Underweight & Normal <25	290 (32.4%)	95 (23.3%)	105 (25.3%)
	Overweight BMI 25–29.9	307 (34.3%)	139 (34.2%)	178 (42.9%)
	Obese BMI ≥ 30	298 (33.3%)	173 (42.5%)	132 (31.8%)
Lean Mass (kg)		52.3 (±11.8)	53.2 (±11.0)	48.7 (±10.8)
Fat Mass (kg)		28.3 (±10.8)	28.9 (±11.3)	26.3 (±8.9) **
BMD (g/cm^2^)		1.16 (±0.12)	1.22 (±0.12)	1.14 (±0.11)
Calcium intake (mg/dL)		1983.0 (±973.1)	1581.7 (±793.0)	1831.7 (±887.1) **
Smoking				
	Current Smoker	223 (24.9%)	95 (23.3%)	70 (16.9%)
MVPA min per week		152.6 (±121.9)	121.0 (±107.7)	153.9 (±122.6) *
**(c)**				
		**20–44 years**	**45+ years**	
**Variable**		***n* = 744 (43.3%)**	***n* = 973 (56.7%)**	
Age		33.0 (±7.1)	56.6 (±7.3) **	
Sex				
	Female	342 (46.0%)	477 (49.0%)	
	Male	402 (54.0%)	496 (51.0%)	
Race/Ethnicity				
	White	344 (46.2%)	551 (56.6%)	
	Black	172 (23.1%)	235 (24.2%)	
	Mexican American	228 (30.7%)	187 (19.2%)	
Height (cm)		169.7 (±9.9)	168.2 (±9.6) *	
Weight (kg)		80.5 (±17.4)	82.1 (±17.4) **	
BMI (kg/m^2^)		27.9 (±5.6)	29.0 (±5.7)	
	Underweight & Normal <25	245 (32.9%)	245 (25.2%)	
	Overweight BMI 25–29.9	269 (36.2%)	355 (36.5%)	
	Obese BMI ≥ 30	230 (30.9%)	373 (38.3%)	
Lean Mass (kg)		52.3 (±11.6)	51.2 (±11.3) *	
Fat Mass (kg)		26.4 (±10.3)	29.2 (±10.5) **	
BMD (g/cm^2^)		1.20 (±0.10)	1.15 (±0.13) **	
Calcium intake (mg/dL)		1965.4 (±1018.3)	1764.1 (±839.7) **	
Smoking				
	Current Smoker	163 (21.9%)	225 (23.1%)	
MVPA min per week		177.0 (±120.6)	121.3 (±113.0) **	

BMD, bone mineral density; BMI, body mass index; min, minutes; MVPA, moderate to vigorous physical activity. Comparisons through ANOVA analysis with Bonferroni correction, with * *p*-value < 0.05. ** *p*-value < 0.001.

**Table 2:** Regression analysis models examining the associations between bone mineral density (g/cm^2^) and other variables.

		Model 1 *			Model 2 **			Model 3 ***		
		R^2^ = 0.33			R^2^ = 0.35			R^2^ = 0.37		
		Beta	SE	*p*-Value	Beta	SE	*p*-Value	Beta	SE	*p*-Value
BMD g/cm^2^	Fat Mass (kg)	−0.004	0.0003	<0.001	−0.003	0.0004	<0.001	−0.005	0.0011	<0.001
	Lean Mass (kg)	0.008	0.0004	<0.001	0.007	0.0005	<0.001	0.004	0.008	<0.001
	Age	−0.03	0.0004	<0.001	−0.034	0.005	<0.001	−0.109	0.023	<0.001
	Sex	0.066	0.0101	<0.001	0.046	0.0103	<0.001	−0.005	0.016	0.75
	Race/Ethnicity	-	-	-	0.017	0.002	<0.001	0.017	0.002	<0.001
	MVPA (mins/wk)	-	-	-	0.00005	0.00002	0.03	0.00004	0.00002	0.05
	Height (cm)	-	-	-	−0.0001	0.0004	0.80	−0.00004	0.0004	0.92
	Smoking Status	-	-	-	0.006	0.006	0.31	0.008	0.006	0.17
	Calcium Intake (mg/dL)	-	-	-	0.0000005	0.000003	0.85	0.0000007	0.000003	0.79
	Fat Mass*Sex	-	-	-	-	-	-	0.002	0.0005	<0.001
	Fat Mass*Age	-	-	-	-	-	-	−0.001	0.0005	0.005
	Lean Mass*Age	-	-	-	-	-	-	0.002	0.0004	<0.001

BMD, bone mineral density. * Adjusted for age and sex. ** Model 1 and additionally adjusted for demographic covariates (race, height (cm)) and behavioral covariates (calcium intake, minutes in MVPA, and smoking status)—main model. *** Model 2 and significant interaction terms.

**Table 3:** Stratified regression analysis models examining the associations between bone mineral density (g/cm^2^) and fat mass (kg) and lean mass (kg), by sex and age categories.

			Fat Mass (kg)		Lean Mass (kg)	
			Beta	*p*-Value	Beta	*p*-Value
BMD g/cm^2^	Age categories *	20–44 years	−0.002	<0.001	0.006	<0.001
		45+ years	−0.005	<0.001	0.009	<0.001
		Menopausal Females	−0.001	0.22	0.005	0.001
	Sex *	Male	−0.005	<0.001	0.008	<0.001
		Female **	−0.002	<0.001	0.006	<0.001
	Race/Ethnicity *	White	−0.003	<0.001	0.006	<0.001
		Black	−0.003	<0.001	0.007	<0.001
		Mexican American	−0.003	<0.001	0.007	<0.001

BMD, bone mineral density. * Stratification analysis utilized model 2. ** Adjusted for demographic covariates (age, sex, race, and height (cm)), and for behavioral covariates (calcium intake, minutes in MVPA, and smoking status), and additionally adjusted for menopause status.

## Data Availability

National Health and Nutrition Examination Survey (NHANES) data are available here: https://wwwn.cdc.gov/nchs/nhanes/ (accessed on 24 November 2019).
